# Development of Swimming Abilities in Squid Paralarvae: Behavioral and Ecological Implications for Dispersal

**DOI:** 10.3389/fphys.2018.00954

**Published:** 2018-07-23

**Authors:** Erica A. G. Vidal, Louis D. Zeidberg, Edward J. Buskey

**Affiliations:** ^1^Center for Marine Studies, University of Parana, Pontal do Paraná, Brazil; ^2^School of Natural Sciences, Chapman Science Academic Center, California State University, Monterey Bay, Seaside, CA, United States; ^3^Marine Science Institute, The University of Texas at Austin, Port Aransas, TX, United States

**Keywords:** cephalopod, *Doryteuthis opalescens*, Reynolds number, swimming behavior, schooling, social interaction, starvation

## Abstract

This study investigates the development of swimming abilities and its relationship with morphology, growth, and nourishment of reared *Doryteuthis opalescens* paralarvae from hatching to 60 days of age. Paralarvae (2.5–11 mm mantle length – ML) were videotaped, and their behavior quantified throughout development using computerized motion analysis. Hatchlings swim dispersed maintaining large nearest neighbor distances (NND, 8.7 ML), with swimming speeds (SS) of 3–8 mm s^-1^ and paths with long horizontal displacements, resulting in high net to gross displacement ratios (NGDR). For 15-day-old paralarvae, swimming paths are more consistent between jets, growth of fins, length, and mass increases. The swimming pattern of 18-day-old paralarvae starved for 72 h exhibited a significant reduction in mean SS and inability to perform escape jets. A key morphological, behavioral, and ecological transition occurs at about 6 mm ML (>35-day old), when there is a clear change in body shape, swimming performance, and behavior, paths are more regularly repeated and directional swimming is evident, suggesting that morphological changes incur in swimming performance. These squid are able to perform sustained swimming and hover against a current at significantly closer NND (2.0 ML), as path displacement is reduced and maneuverability increases. As paralarvae reach 6–7 mm ML, they are able to attain speeds up to 562 mm s^-1^ and to form schools. Social feeding interactions (kleptoparasitism) are often observed prior to the formation of schools. Schools are always formed within areas of high flow gradient in the tanks and are dependent on squid size and current speed. Fin development is a requisite for synchronized and maneuverable swimming of schooling early juveniles. Although average speeds of paralarvae are within intermediate Reynolds numbers (Re < 100), they make the transition to the inertia-dominated realm during escape jets of high propulsion (Re > 3200), transitioning from plankton to nekton after their first month of life. The progressive development of swimming capabilities and social interactions enable juvenile squid to school, while also accelerates learning, orientation and cognition. These observations indicate that modeling of the lifecycle should include competency to exert influence over small currents and dispersal patterns after the first month of life.

## Introduction

The California market squid, *Doryteuthis opalescens* (Berry, 1911), is an important fishery resource and a key forage species that lives in the nearshore pelagic environment. The species has complex spawning behavior laying benthic eggs that incubate for 3–9 weeks depending on temperature. Upon hatching, planktonic paralarvae are negatively geotactic (Sidie and Holloway, 1999), passive drifters and become entrained within 3 km of shore by interacting with tidally reversing currents and cyclonic gyres. Paralarvae perform vertical diel migration (Zeidberg and Hamner, 2002), and their greatest abundance is associated with cooler sea surface temperatures (SST) during La Niña events (Van Noord and Dorval, 2017). Juveniles are nektonic, live on the shelf (Zeidberg, 2014) and move to the slope or over deep water with development. Adults return to the shelf to aggregate into spawning groups of millions of individuals, where they are the focus of the largest cephalopod fishery in the United States (Ryley, 2015).

Squids are highly active social animals and form schools at an early-life stage (Sugimoto and Ikeda, 2012; Vidal and Boletzky, 2014). Social behavior and the ability to gather in schools where individuals synchronize to each other’s SS in parallel orientation (polarization) is a major ecological and behavioral adaptation, as it requires complex swimming and cognitive skills (Hurley, 1978; Sugimoto and Ikeda, 2012; Sugimoto et al., 2013; Yasumuro et al., 2015). The underlying mechanisms allowing squid to make the transition from passive planktonic drifters to active schoolers early in life have received little attention. Schooling is believed to provide many benefits to individuals by improving foraging strategies, reducing predation risk, accelerating learning, and improving orientation skills (Masuda et al., 2003; Oshima et al., 2016).

Throughout their life cycle, squid use a dual mode system for swimming, combining pulsed jetting and fin-flapping (Bartol et al., 2008, 2016; Stewart et al., 2010). However, the hydrodynamic efficiency of this dual mode locomotive system changes considerably during ontogeny due to changes in relative size and shape of the body and fins, mantle muscle structure, and flow regimen (Hoar et al., 1994; Thompson and Kier, 2001; Bartol et al., 2008, 2016; Thompson et al., 2010). Paralarvae experience intermediate Re, a fluid regime in which both viscous and inertial flow forces have important effects, compared with the high Re regime of adult animals. Hatchlings swim predominantly using high-volume, low-velocity pulsed jets as they have rudimentary fins and larger relative funnel apertures, moving proportionately a greater distance with each jet (Bartol et al., 2008, 2009; Staaf et al., 2014). It was shown that the propulsive efficiency (thrust) of the exhalant phase of the jet was significantly greater in newly hatched *Doryteuthis pealeii* than in adults (Bartol et al., 2008, 2009). Little has been reported, however, on the progressive improvement of these swimming abilities during early ontogeny. In adults, the jet propulsive efficiency improves at higher SSs (Anderson and Grosenbaugh, 2005) and fins have a fundamental role in generating thrust (Anderson and Demont, 2005), stabilizing the body, and also providing net lift (Stewart et al., 2010; Olcay and Malazi, 2016).

Understanding how swimming abilities develop during early ontogeny in squid is of paramount importance to evaluate the extent to which active swimming influences dispersal, distribution, and sizes of a population as the environment changes from year to year. Dispersal is recognized as a consequence of planktonic development (Scheltema, 1986). It was recently demonstrated that the developmental mode (planktonic or benthic) of cephalopods influences their dispersal ability to such an extent that it can determine the broader distributional range of species with planktonic hatchlings (Villanueva et al., 2016). As paralarvae develop, the interplay between their swimming abilities, behavior, and the local currents dictates distribution, dispersal or retention, growth, and survival.

The end of the planktonic dispersive phase will be determined by the ability of paralarvae to hold a position against a current (sustained swimming) and to form schools. This ability should be regulated by morphology and size and ultimately nourishment. Starvation is considered one of the major regulators of larval growth and survival in the sea (Boidron-Métairon, 1995), and while previous studies have confirmed that *D. opalescens* paralarvae are extremely sensitive to starvation (Vidal et al., 2002b, 2006), little work has been directed toward understanding the effects of food availability on the swimming performance of paralarvae. More precisely, how does food availability impact paralarvae swimming ability and pattern? What are the main developmental processes and morphological attributes that allow squid to transition from plankton to nekton and to swim in schools?

Upon hatching, *D. opalescens* is dispersed by currents and relies on jet-and-sink swimming, but within the first 2 months of life they develop the ability to form schools. We used motion analysis combined with morphological and growth data from reared *D. opalescens* paralarvae to provide an assessment of swimming performance and uncover interconnected ecological and behavioral milestones. Specifically, this study sought to(1) examine the development of swimming abilities of paralarvae and their interplay with growth, morphological attributes, and behavior, (2) evaluate the effects of starvation on the swimming performance of paralarvae, and (3) investigate how and when squid achieve the transition from plankton to nekton.

## Materials and Methods

### Field Collection and Experiments

Eggs of *D. opalescens* were collected by SCUBA divers on the spawning grounds (15–30 m) in Monterey Bay (36°60′N, 121°80′W) and Southern California (34°7′N, 119°05′W), United States. After collection, the eggs were placed in sealed plastic bags with seawater, filled with pure O_2_, and air-shipped in a cooler box with frozen ice packs to the National Resource Center for Cephalopods, University of Texas Medical Branch, Galveston, TX, United States, where three experiments took place. In all experiments, eggs and paralarvae were reared at 16 ± 0.5°C in a recirculating system consisting of seven 220-l cylindrical tanks (0.95 m diameter × 0.4 m height). The water inflow of the rearing tanks was maintained at a rate of 5.7 l min^-1^ generating a counter-clockwise current of ∼1 cm s^-1^ that promoted an even distribution of the paralarvae and their prey (Vidal et al., 2002b).

The number of paralarvae per tank ranged from 800 to 3000 and the food offered was *Artemia* spp. nauplii enriched with SUPER SELCO (INVE^®^), mysid shrimp (*Americamysis almyra*), and wild zooplankton (mainly copepods) at densities of 50–200 prey l^-1^ (Vidal et al., 2002b). In the starvation experiment, paralarvae were kept in the hatching tank up to day 14 after hatching when a total of 2100 were randomly transferred to three other tanks (700 paralarvae in each tank) and exposed to different periods of starvation. The first day of the experiment was day 15 after hatching and each experimental tank constituted an experimental group that was exposed to 24, 48, and 72 h of starvation, for the purpose of comparing the swimming performance of fed paralarvae with those exposed to one, two, and three days of starvation.

### Video Recording of Behavior

During the experiments, paralarvae ranging in size from 2.5 to 11 mm ML were filmed between 14:00 to 20:00 h in the rearing tanks at 0, 5, 15, 16, 17, 18, 40, 50, and 60 days after hatching. In total, 17 h of filming was performed, from 1 to 2 h for each age. Videos were recorded with a Sony CCD-TR930 Hi8 camcorder operating at 30 frames s^-1^ fitted with a #1.5 close-up lens. The camera was mounted on a tripod at a 90° angle to the glass window on the side of the tanks, the frame of view for filming was 3.6 cm × 3.6 cm. Distance calibration was performed prior to each filming session. A small thin ruler was positioned inside the tanks facing the window. The camera was set to operate in manual mode and the autofocus and zoom functions were turned off, then the focus of the camera was adjusted to the ruler with a focal distance 1–3 cm in toward the center of the tank from the window. The lens aperture was also locked to maintain a constant depth of field (3 mm). Paralarvae were videotaped when they came into the frame of view and were in focus for the small depth of field. Errors resulting from the positioning of the paralarvae along the optical axis were estimated to be below 15% for hatchlings and decreased as paralarvae increased in size. During filming, the camera was connected to a TV set to allow monitoring of behavior without disturbing the paralarvae.

The water inflow of the tanks was turned off until the current had reduced to 1 mm s^-1^, and then video-taping occurred for 20 min. The same procedure was repeated in at least three other tanks, holding same aged squid. Current speed was measured with a flowmeter (Flo-Mate, Marsh-McBirney, Frederick, Md, model 2000; Vidal et al., 2002b). Thus, SSs could be underestimated by up to 1 mm s^-1^, but this effect would be similar for all ages.

### Paralarvae Swimming Speed and Behavior by Motion Analysis

To describe the swimming pattern and behavior of *D. opalescens*, paralarvae video recordings of 0-, 5-, 15-, 40-, 50-, and 60-day-old-fed paralarvae and of 16-, 17-, and 18-day-old starved paralarvae were played back through a Motion Analysis VP-110 video-to-digital processor, and digitized outlines of the paralarvae were sent to a computer at a rate of 15 or 30 frames s^-1^, depending on the mean displacement SS of the paralarvae. Slower swimming paralarvae (younger) were sampled at 15 frame s^-1^, but faster swimming paralarvae (older) were sampled at the full 30 frames s^-1^, for higher temporal resolution. Approximately 10 min of combined swimming behavior from at least 15 paralarvae of each age filmed were examined (∼9000 measurements at 15 frames s^-1^; Buskey et al., 1993). The swimming patterns were quantified in a two-dimensional representation of a three-dimensional swimming pattern; thus, if a paralarva was swimming toward or away from the camera, it would appear stationary; however, these errors were reduced by focusing the camera to a shallow depth of field, and only using images that were in focus. Thus, only trajectories perpendicular to the camera were precisely recorded.

Paths of movement and motion parameters were calculated using Expertvision Cell-Track computer software. Mean SS (mm s^-1^) and mean RCDs (° s^-1^) were calculated from paths of movements during each interval. RCD is an index of turning rate irrespective of direction, and is measured as the absolute value of the angular velocity. The change in both *X* and *Y* positions were used to measure the displacement between two central locations for the paralarvae over a known time interval and this change in location over time was used to calculate speed. The tendency of paralarvae to remain within an area by changing their turning behavior was indicated by the mean NGDRs of successive cumulative segments of their paths of travel. NGDR is an index of path tortuousness or convolution; a ratio of net displacement (the linear distance between starting and ending points of a path) to gross displacement (the total distance traveled by the path over the same time interval). Basically, a straight path gives an NGDR value of 1 and a closed circular path a value of 0. This ratio was measured repeatedly, as the swimming path lengthened over time. Mean parameters are based on multiple paths from several paralarvae of each age. Mann–Whitney–Wilcoxon tests were used to compare SS, NGDR, and RCD, according to Zar (1996).

### Mantle Length, Fin Width, and Dry Weight Measurements

Random samples of 5–10 paralarvae were collected from the rearing tanks for each age after recording swimming behavior. Sampled paralarvae were anesthetized with magnesium chloride and both ML and FW were measured according to Roper and Voss (1983) to the nearest 0.01 mm under a dissecting microscope equipped with an ocular micrometer. Then, dry body mass (dry weight – DW) were obtained individually from 5 to 10 paralarvae after placement in an oven at 60 °C for 24 h, using a microbalance to the nearest 0.01 mg (as in Vidal et al., 2002a). Also, samples of 5–10 other age paralarvae (0-, 1-, 2-, 3-, 4-, 6-, 7-, 10-, 11-, 13-, 17-, 19-, 23-, 24-, 25-, 27-, 31-, 33-, 35-, 37-, 42-, 45-, 47-, and 55-day-old) were sampled to obtain a more precise relationship between DW and ML and DW and age.

### Length–Weight Relationship and Length–Fin Width Relationship

The regression of the length–weight relationship was calculated from dry weight of paralarvae versus ML as in the formula:

$$DW={aML}^{b}$$

where DW is dry weight, ML is mantle length, *a* is a constant, and *b* the allometric factor. DW and ML were log transformed to produce a linear relationship, and then *a* and *b* were estimated by least square regression. The relative growth between FW and ML was analyzed by the allometric equation:

$$FW={aML}^{b}$$

where *b* is the allometric constant and *a* is the initial index. After logarithmic transformation of *FW* and *ML*, *a* and *b* were estimated by least square regression. Growth relationship between the two linear variables indicate negative allometry when *b* < 1, showing that FW grows less rapidly than the ML, positive allometry when *b* > 1, and isometry when *b* = 1. A significance test for comparison of the slope against 1 was applied (Sokal and Rohlf, 1995).

### Survival Rates and Growth Relationships

Mortality was determined daily by counting the number of dead paralarvae in each replicate tank during the experiments. Survival was calculated as the percentage of live paralarvae left in each replicate tank versus the initial number, excluding the paralarvae sampled for data collection. Final survival rates were expressed as the minimum and maximum values obtained from all the replicate tanks in each experiment.

Growth rates were expressed as instantaneous GRs and were calculated using the standard exponential function:

$$Y=Y_{1}e^{bd}$$

where *Y* is the mean body DW or ML, *Y*_1_ is the mean DW or ML obtained on hatching day, *e* is the natural logarithm, *b* is the slope, and *d* is age in days post-hatching. The instantaneous relative GRs expressed in % body DW day^-1^ and in % ML day^-1^ were calculated using the formula: GR = 100 × (*eb*^-1^). Both age–weight and age–size relationships were fitted to the exponential equation. The time required for a squid to double its weight or size (doubling time) was calculated by dividing the natural log of 2 by the GR value obtained based on the hatching weight and that at 60 days (Forsythe and Hanlon, 1988).

### Fin Beats, Mantle Width, Swimming Angle Measurements, and Reynolds Number Calculations

Fin beat frequency and MW were measured from image analysis of 15–25 paralarvae of 0, 15, 40, and 60 days of age. These measurements were captured from a video camera positioned above a small round aquarium (7 cm H, 33 cm diameter) that was a miniature model of the rearing tanks. The MW measurements were obtained according to Roper and Voss (1983) from still frame images to evaluate morphological changes in the mantle with development. During filming, a scale was positioned at the bottom of the aquarium to set the scale for each image and distance calibration was performed prior to filming as described above. Measurements were performed by digitizing a line on the image, and the width values were stored.

**FIGURE 1:**
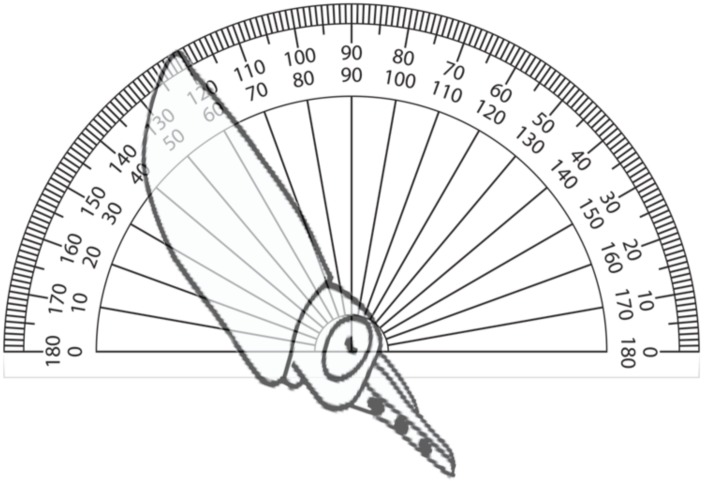
Diagram of angular orientation of paralarvae. Angles of orientation were measured by comparing the horizon to a line drawn from the eye lenses and the posterior tip of the mantle of paralarvae as reference points. The angle shown is 60°.

To evaluate schooling behavior, the angular orientation and the NND were measured from filming performed through the window of the rearing tanks. A protractor was used to obtain the angular orientation by comparing the horizon to a line drawn from the eye lenses and the posterior tip of the mantle of paralarvae as reference points (**Figure 1**). The NND was measured as the distance between the eyes of randomly selected paralarvae and was standardized to the mean ML of measured squid. Both the angles and the NND were obtained from 5 to 22 paralarvae of each age. To ensure precision and accuracy of measurements, squid were only measured when within a predefined distance and orientation to the camera, when their eyes were exactly parallel to the video camera and in focus. *T*-tests were used to compare the orientation angles and NND (Sokal and Rohlf, 1995).

The MLs were used to calculate Re, using the following equation:

Re⁡=UL/v

where *L* is ML in meters, *U* is swimming velocity obtained from motion analysis (see above) in m s^-1^, and *v* is the kinematic viscosity of water. The kinematic viscosity, *v*, is 1.155614E-06 m^2^ s^-1^ in 16°C seawater of 33 g kg^-1^ (Sharqawy et al., 2010). Small paralarvae yield lower intermediate Re values indicating that the effects of viscosity on swimming performance are significant.

### Ethics Statement

This study has been conducted in compliance with recommendations of the ARRIVE Guideline (Kilkenny et al., 2010) for reporting *in vivo* experiments with research animals. The Institutional Animal Care and Use Committee (IACUC) of the University of Texas did not require researchers to submit protocols for the ethical treatment of invertebrate larvae at the time this research was performed.

## Results

### Swimming Performance, Paths, and Speeds of Fed Paralarvae

**FIGURE 2:**
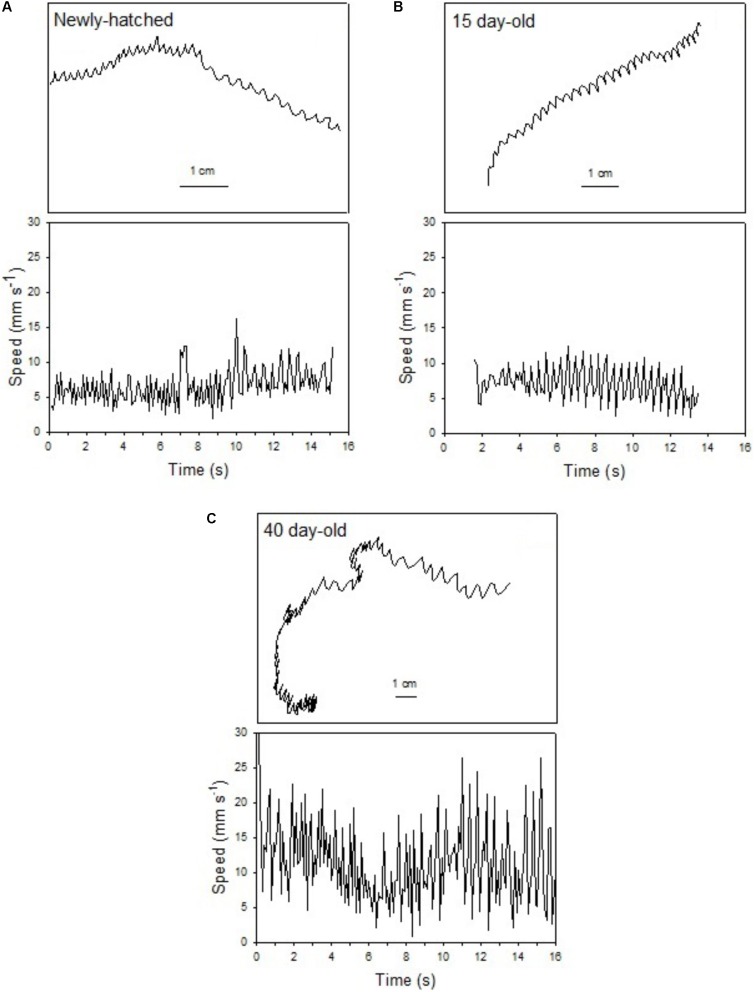
*Doryteuthis opalescens.* Representative swimming path records in the vertical plane and swimming speed patterns (14–16 s duration) of fed paralarvae. **(A)** Newly hatched, **(B)** 15-day-old, and **(C)** 40-day-old. Paralarva is traveling from left to right as recording time increases.

**FIGURE 3:**
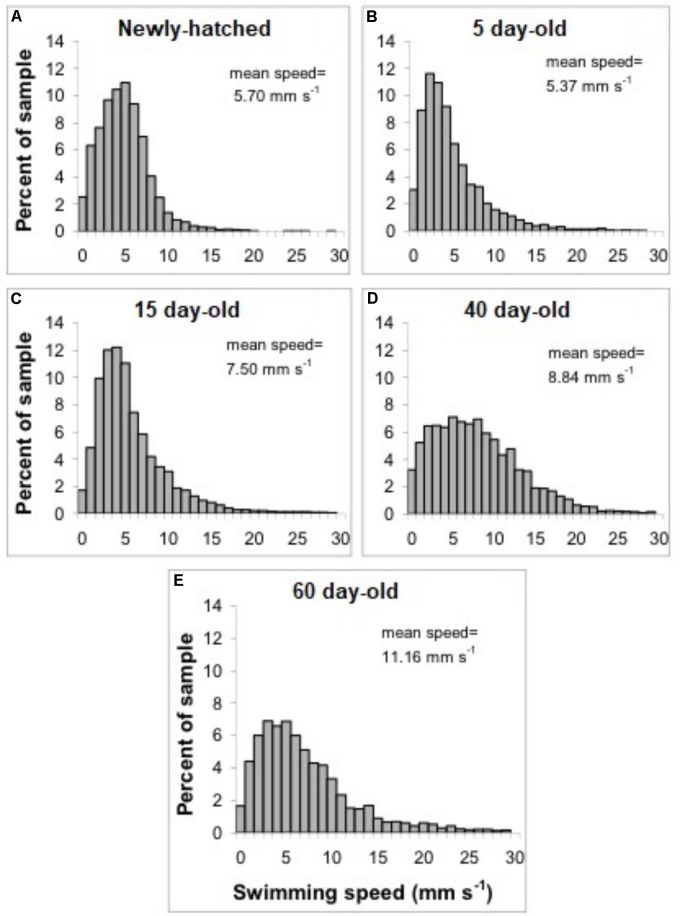
*Doryteuthis opalescens.* Distribution of swimming speeds of paralarvae. **(A)** Newly hatched, **(B)** 5-day-old, **(C)** 15-day-old, **(D)** 40-day-old, and **(E)** 60-day-old.

The swimming of a newly hatched squid with a mean ML of 2.65 mm was characterized by the predominance of a short pulsed jet-and-sink motion (vertical bobbing). This resulted from mantle contractions causing jetting up and mantle expansion and negative buoyancy causing sinking. The typical swimming paths showed large speed variation, with frequent change from low speed to several peaks of higher speed in time intervals of 0.1–0.2 s (**Figure 2A**). These changes took place within a narrow speed range (4–8 mm s^-1^) with mean SS of 5.70 mm s^-1^ (**Figures 2A**, **3A**). Hatchlings drift with the current and showed paths with relatively long horizontal displacements and short excursion in the vertical plane, resulting in high NGDR values and mean RCD around 300° s^-1^. The relative SS was 2.2 ML s^-1^ and the Re was 13 (**Table 1**). The swimming paths observed for 5-day-old paralarvae were very similar to those of newly hatched paralarvae, but there was more variance in speed (**Table 1** and **Figure 3B**).

**Table 1:** Doryteuthis opalescens.

Age (d)	ML (mm)	Swimming speed (mm s^-1^)	Maximum speed (mm s^-1^)	ML s^-1^	Fin beats s^-1^	NGDR	RCD (° s^-1^)	Reynolds number swimming speed	Reynolds number maximum speed
0	2.65 ± 0.07	5.70 ± 0.4^a^	67	2.2	2.8 ± 0.85	0.63 ± 0.10^a^	298 ± 47^a^	13	154
5	2.70 ± 0.13	5.37 ± 0.7^a^	84	2.0	–	0.64 ± 0.07^a^	297 ± 70^a^	13	196
15	3.78 ± 0.24	7.50 ± 1.5^b^	208	2.0	3.6 ± 0.47	0.71 ± 0.04^a^	275 ± 21^a^	26	680
40	6.70 ± 0.92	8.84 ± 1.6^b^	562	1.3	5.1 ± 1.76	0.36 ± 0.07^b^	564 ± 107^b^	51	3258
50	7.23 ± 1.15	6.16 ± 1.6^a,b^	–	0.9	–	0.37 ± 0.08^b^	571 ± 89^b^	39	–
60	9.81 ± 1.41	11.16 ± 3.0^b^	–	1.2	6.5 ± 3.06	0.36 ± 0.05^b^	586 ± 68^b^	95	–


*Mantle lengths (ML), swimming speed, net to gross displacement ratio (NGDR), and rate of change of direction (RCD) of paralarvae reared from 0 to 60 days after hatching. Values are means of 5–15 individuals ± SD. Means with same superscript letters denote no statistical difference (Mann–Whitney–Wilcoxon test, *p* > 0.05). Reynolds numbers are calculated from the mean speed (swimming or maximum) *U*, ML, and *v*, the kinematic viscosity of seawater at 16°C and 33 g kg^-1^ (Sharqawy et al., 2010), Re = *UL/v*.*

In 15-day-old paralarvae (3.78 mm ML), the swimming paths exhibited longer and more consistent time intervals between jets (**Figure 2B**). These paralarvae showed enhanced activity, with more variation in speed and a significantly higher mean speed (7.50 mm s^-1^), than newly hatched and 5-day-old paralarvae (*p* < 0.05, **Table 1**) and maximum speeds of 208 mm s^-1^ (**Figures 2B**, **3C**). The NGDR and RCD were not significantly different from early paralarvae, but Re numbers were fourfold higher for maximum speed when compared to hatchlings (**Table 1**).

As paralarvae reached 40 days of age and 6–7 mm ML, they were able to perform very fast changes of speed, accelerating from 5 to 50 mm s^-1^ in approximately 0.2 s (**Figure 2C**). The path displacement was more circuitous when compared with 15-day-old paralarvae. Due to more powerful jets, the distance traveled by paralarvae during vertical displacement was larger than the linear distance between the start and ending points of the paths (**Figure 2C**). This resulted in significantly lower NGDR values (*p* < 0.05, **Table 1**), half of those measured for early paralarvae (**Table 1**). Mean RCD was higher (*p* < 0.05) and doubled in paralarvae from 15 to 40 days. This was a reflection of considerable changes in the swimming pattern, as older squid spent more time hovering. By doing so, they remain in the same area for longer periods of time by maneuvering and adjusting their orientation to other squid. Mean speed was 8.84 mm s^-1^ with greater variance (**Figure 3D**), and maximum speeds reached 562 mm s^-1^. The relative SS decreased to 1.3 ML s^-1^, due to hovering.

Paralarvae of 6–7 mm ML were able to jet in all directions at much higher speeds than 15-day-old paralarvae, performing faster horizontal displacements, both during predatory (jetting forward) and escape behavior (jetting backward). A more regularly repeated (cyclic) SS pattern became evident during jetting due to hovering, with short periods (∼5 s) jetting at mean speed of 15 mm s^-1^, interspersed with other periods of jetting at 8 mm s^-1^ (**Figure 2C**). The Re for maximum speed quadrupled between days 15 and 40, demonstrating that 40-day-old squid are able to attain a wide range of speeds and Re in the inertial realm (**Table 1**).

The swimming paths observed for 60-day-old squid (9.81 mm ML) were similar to those of 40-day-old squid (**Figure 2C**), but reflected enhanced maneuverability, stability and jetting control (**Table 1**). Squid spent most of the time hovering; therefore, the relative path displacement was greatly reduced. The mean SS was 11.16 mm s^-1^ and the distribution of speeds was strongly skewed to the right, showing the capacity for attaining and maintaining higher speeds (**Table 1** and **Figure 3E**). Due to the relatively small field of view when compared to the total size of 60-day-old squid, it was not possible to document maximum speed.

### Swimming Behavior and Social Interactions of Fed Paralarvae

**FIGURE 4:**
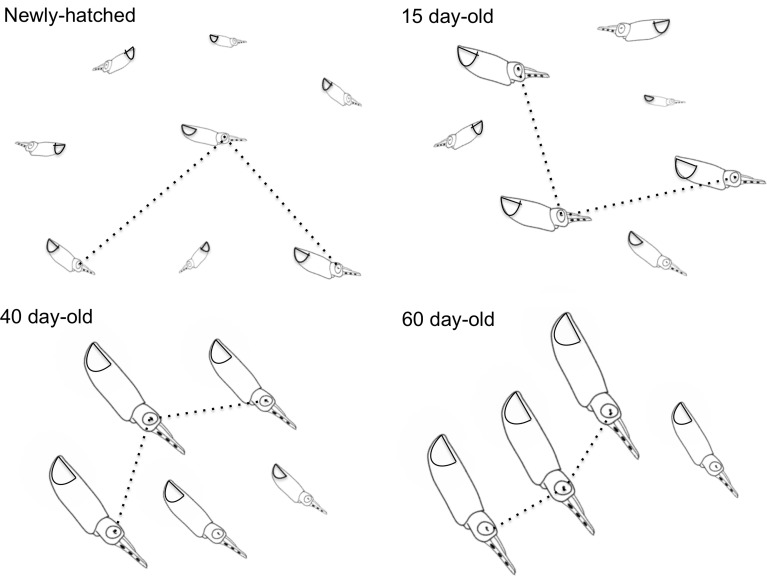
*Doryteuthis opalescens.* Stereotyped swimming behavior patterns of paralarvae reared for 60 days after hatching. The dotted lines indicate the nearest-neighbor distance (NND) measured as the distance between the eyes of randomly selected paralarvae.

**FIGURE 5:**
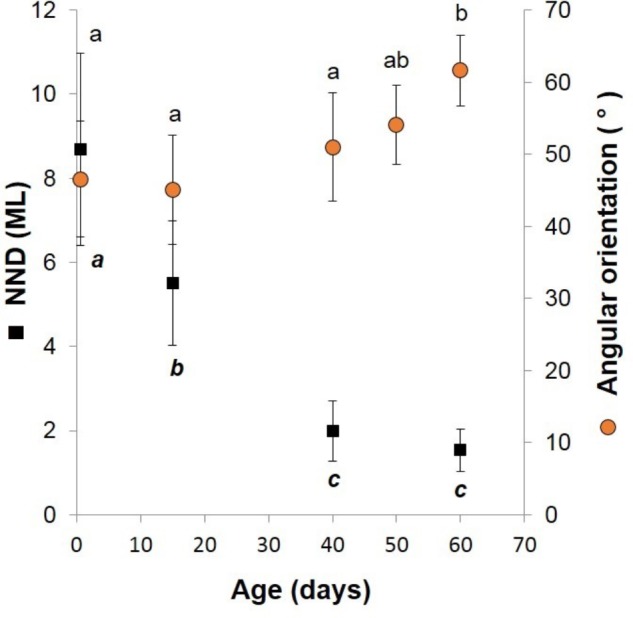
*Doryteuthis opalescens*. Relationships between the nearest-neighbor distance (NND) and swimming angles of orientation and age in squid reared for 60 days after hatching. The NND was measured as the distance between the eyes of randomly selected paralarvae and was standardized to the mean ML of measured squid. Symbols and bars indicate mean and SD, respectively; means with same superscript letters denote no statistical difference (*t*-test, *p* > 0.05).

**FIGURE 6:**
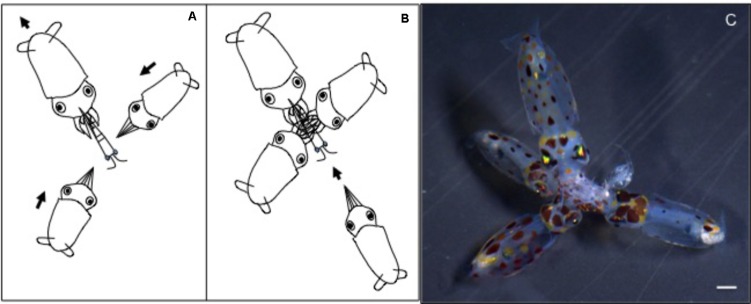
*Doryteuthis opalescens.* Stereotyped group feeding behavior of paralarvae as a result of kleptoparasitism, a competition involving stealing of already-captured prey in possession of another paralarvae (host). **(A)** A large paralarva captures a large prey (mysid shrimp), which cannot be enclosed within the arms. **(B)** Other smaller paralarvae attack the captured prey. **(C)** Several paralarvae feed on the same prey.

Newly hatched paralarvae had internal yolk sacs that occupy a relatively large portion of the mantle cavity. Hatchlings swam in random directions, dispersed and maintaining a mean distance of 8.7 ML from each other by jetting and sinking continuously, oriented at 45° angles (**Figures 4**, **5**). They drift with the current and the short horizontal displacements against the current were primarily due to prey inspection and attack, when fins were mainly used, beats average 2.8 s^-1^ (**Table 1**). Hatchlings often displayed aggressive behavior toward one another, chasing and attacking other paralarvae that swam nearby with knocks of arms and tentacles tips. Often after a paralarva had captured a large prey (mysid shrimp) relative to its size, the prey was attacked by several other paralarvae (up to 7) and all feed on the same prey (**Figure 6**). This kleptoparasitism is intensified 6–7 days after hatching, when yolk reserves are fully absorbed.

Fifteen-day-old paralarvae still swam randomly, although they were observed more often in the same angular orientation (**Figure 4**), maintaining a mean distance from one another of 5.5 ML (**Figure 5**). They were able to swim up and down in the water column easily, and more often horizontally, both with and against the current. Horizontal displacements were often related to prey inspection, pursuit, and attacks. During the phase of positioning prior to a prey attack, paralarvae remained quasi-stationary by means of rapid fin beats, averaging 3.62 beats s^-1^ (**Table 1**). Kleptoparasitism remained frequent.

Between 30 and 40 days of age, paralarvae showed a considerable change in swimming behavior, holding position against the current, and swimming close to each other. Concomitantly, aggressive behavior, toward other squid swimming nearby was reduced. Kleptoparasitism also was reduced likely because prey size relative to squid size diminished. Schooling behavior is defined here as a group of at least three squid swimming in the same direction in parallel orientation and positioned within one to three ML of each other (Pitcher and Parrish, 1993; Sugimoto and Ikeda, 2012). Schooling was first observed in 35- to 45-day-old paralarvae (6.0–8.0 mm ML). The largest paralarvae in the tanks were the first to swim against the current and to form schools. The size of the school progressively increased from 5 to 15–40 squid and they were always formed in the same place, close to the surface, against the current and underneath the water inlet spray bar, where the current was at its maximum velocity. Sometimes, more than one school could be seen in a tank, but each school was sorted by size. The smallest paralarvae in the tanks did not take part in the schools, as they were not able to hold a position against the current. Although the same age as the squid forming schools, these paralarvae swam randomly close to the surface. The larger schooling squid often vigorously attacked small squid that approached the school.

Forty-day-old schooling squid spent most of the time hovering significantly closer to one another (2.0 ML; **Table 1** and **Figures 4**, **5**). Nevertheless, they were able to swim horizontally both forward (arms first) and backward (tails first) crossing the tank (0.8 m) in fractions of a second (0.2 s). Forward horizontal displacements were mainly caused by interactions with prey or with other squid, during which fins utilization increased, especially during the positioning phase prior to prey capture; fin beats averaged 5.12 beats s^-1^ (**Table 1**).

Sixty-day-old schooling squid showed enhanced swimming control and parallel orientation (polarization) when compared with 40-day-old squid. They swam in a more vertical orientation (62°) with mean fin beats of 6.51 beats s^-1^ (**Table 1** and **Figure 5**). These skilled movements permitted swimming closer to other squid, resulting in mean NND of 1.5 ML (**Figures 4**, **5**), and the highest level of synchrony and fine-scale movements powered by fins and jet propulsion. This synchrony was particularly evident when any disturbance occurred, as for example, a shadow from an observer or when any squid in the school changed orientation or speed in an escape reaction. Immediately after such disturbances, the squid responded similar to a flash explosion, scattering from a polarized orientation by jetting backward without colliding and disrupting the school, dispersing briefly and then reassembling.

### Swimming Paths and Speeds, and Behavior of Starved Paralarvae

**FIGURE 7:**
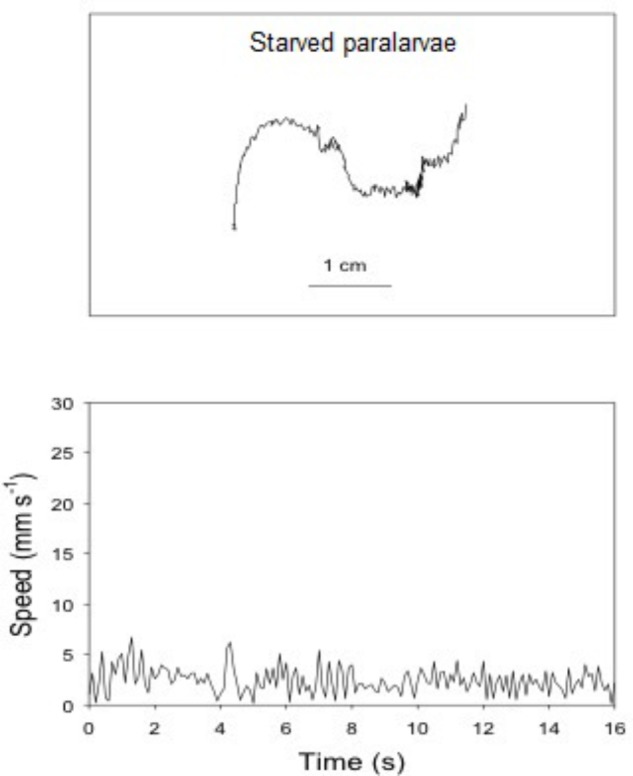
*Doryteuthis opalescens.* Representative swimming path records in the vertical plane and swimming speed patterns (14–16 s duration) of 18-day-old paralarvae starved for 72 h.

**FIGURE 8:**
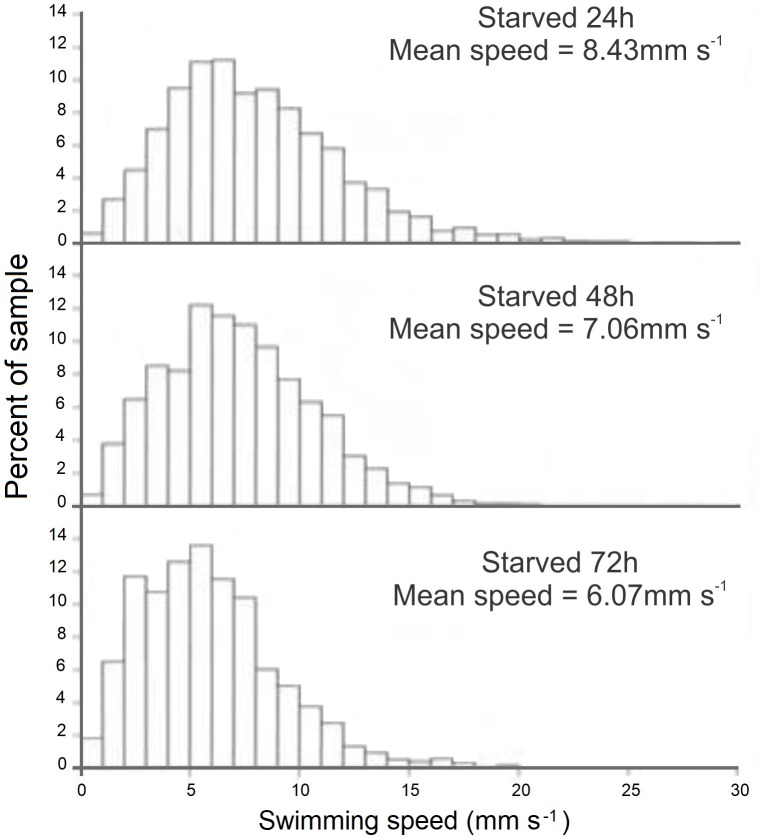
*Doryteuthi*s *opalescens.* Distribution of swimming speeds for 15-day-old paralarvae starved for 24, 48, and 72 h.

The swimming paths of 16-, 17-, and 18-day-old paralarvae starved for 24, 48, and 72 h, respectively, showed an erratic pattern with speeds ranging from nearly 0 to 10 mm s^-1^ (**Table 2**). The distribution of SS of starved paralarvae showed marked changes when compared to that of 15-day-old fed paralarvae. The speed was distributed mainly between 5 and 8 mm s^-1^ slightly skewed to the right (**Figures 7**, **8**). Mean SS of 15-day-old fed paralarvae and 16-day-old paralarvae starved for 24 h were not statistically different (Mann–Whitney–Wilcoxon test, *p* > 0.05; **Figure 8** and **Tables 1**, **2**). A reduction in mean RCD values was also observed, showing that starved paralarvae changed direction less often when compared to 15-day-old fed paralarvae and even to newly hatched squid (**Table 1**, **2**), suggesting decrease in swimming activity. Increasing the starvation period led to a significant reduction in the mean SS of 18-day-old paralarvae and a significant reduction in mean RCD for 16- and 17-day-old starved paralarvae (Mann–Whitney–Wilcoxon test, *p* < 0.05; **Table 2** and **Figures 7**, **8**). Similarly, the NGDR was significantly different between 15-day-old-fed and 16- and 17-day-old starved paralarvae, but not to 18-day-old starved paralarvae.

**Table 2:** *Doryteuthis opalescens*.

Age (d)	Starvation period (h)	ML (mm)	Swimming speed (mm s^-1^)	Maximum speed (mm s^-1^)	ML s^-1^	NGDR	RCD (° s^-1^)
15	–	3.78 ± 0.24	7.50 ± 1.5^a^	208	2.00	0.71 ± 0.04^a^	275 ± 21^a^
16	24	3.91 ± 0.22	8.43 ± 1.8^a^	23	2.16	0.74 ± 0.09^b^	166 ± 30^b^
17	48	3.72 ± 0.35	7.06 ± 1.5^a^	18	1.90	0.81 ± 0.05^b^	192 ± 50^b^
18	72	3.55 ± 0.41	6.07 ± 1.3^b^	18	1.82	0.69 ± 0.09^a^	303 ± 80^a^


*Age, mean mantle lengths (ML), swimming speeds, net to gross displacement ratio (NGDR), and rate of change of direction (RCD) of 15 day-old paralarvae starved for 24, 48, and 72 h. Values are means ± SD and means with same superscript letters denote no statistical difference (Mann–Whitney–Wilcoxon test, *p* > 0.05).*

Sixteen-day-old paralarvae starved for 24 h spent most of the time searching for food. They frequently swam horizontally and were observed to inspect, attack, and often capture small particles that were released immediately after determination as a non-prey item. The arms and tentacles spread out and extended in repeated searching. This behavior had the effect of increasing horizontal displacement. The swimming behavior observed for 17- and 18-day-old paralarvae starved for 48 and 72 h, respectively, changed considerably when compared to 16-day-old paralarvae. The main swimming behavior was the jet and sink of hatchlings, but with even lower speeds (**Figure 8**). These paralarvae showed a lack of interest in particles, and had fewer horizontal movements. Indeed, they moved little, jetting passively and swimming close to the bottom most of the time, rarely close to the surface.

### Survival Rates

Survival rates at day 10 after hatching were between 70 and 78.4%, and decreased to 54.3–67% on day 20. From then on, survival decreased relatively slowly, reaching 48.2–60.7 at day 40. Final survival rates were between 42 and 59% at day 60. The highest mortality was observed during the first 10 days after hatching and coincided with the no net growth phase during first feeding. A second peak of mortality occurred at day 40.

### Relative Growth and Development of Fins

**FIGURE 9:**
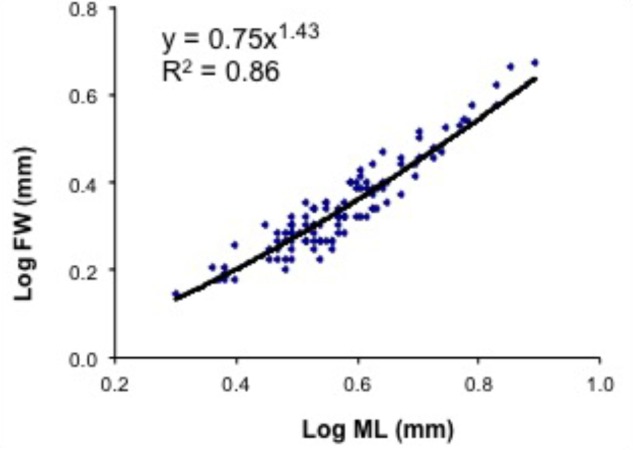
*Doryteuthis opalescens*. Relationship between mantle length (ML) and fin width (FW) in paralarvae reared for 60 days after hatching (*n* = 104).

The relative growth of fins was examined in 104 paralarvae from 2.5 to 8.2 mm ML. In hatchlings, the FW represented from 55 to 72% of ML, but in 40-day-old paralarvae (>6.0 mm ML), the FW/ML decreases to 43% and this ratio increases again to 50% by day 50 (**Figure 9** and **Table 3**). No discontinuity point was detected (*F* = 3.166, *p* = 0.779), and the relationship between FW and ML showed an allometric constant of 1.43 (**Figure 9**). Relative GRs for FW were low soon after hatching (0.52% day^-1^), but increased to 1.21% day^-1^ between 5 and 15 days after hatching and reached even higher rates in 40- and 50-day-old paralarvae, 1.38 and 1.95% day^-1^, respectively, demonstrating that fins grows faster than the ML in paralarvae older than 15 days of age (>4.0 mm ML; **Table 3**).

**Table 3:** *Doryteuthis opalescens*.

Age (d)	ML (mm)	ML growth rates (mm day^-1^)	DW (mg)	DW^(1/3)^/ML	DW growth rates (mg day^-1^)	FW (mm)	FW/ML	FW growth rates (mm day^-1^)	MW (mm)	MW/ML	MW growth rates (mm day^-1^)
0	2.65 ± 0.07	–	0.46 ± 0.07	0.29	–	1.90 ± 0.13	0.72	–	1.92 ± 0.26	0.78 ± 0.07	–
5	2.70 ± 0.13	0.37	0.40 ± 0.06	0.27	–2.69	1.95 ± 0.14	0.72	0.52	2.10 ± 0.55	0.72 ± 0.07	1.81
15	3.78 ± 0.24	3.42	1.21 ± 0.32	0.28	11.70	2.20 ± 0.20	0.58	1.21	2.32 ± 0.29	0.63 ± 0.05	0.98
40	6.70 ± 0.92	2.06	5.40 ± 1.94	0.26	6.17	2.90 ± 0.53	0.43	1.38	2.66 ± 0.73	0.45 ± 0.05	0.55
50	7.23 ± 1.15	1.35	8.20 ± 2.01	0.28	4.27	3.60 ± 0.81	0.50	1.95	–	–	–
60	9.81 ± 1.41	3.17	16.50 ± 2.39	0.26	7.24	–	–	–	–	0.37 ± 0.02	1.11


*Growth rates of paralarvae reared from 0 to 60 days after hatching. Means were obtained from samples of 15 to 40 paralarvae per age. Mantle length (ML), dry weight (DW), fin width (FW), mantle width (MW).*

### Growth in Length and Weight

**FIGURE 10:**
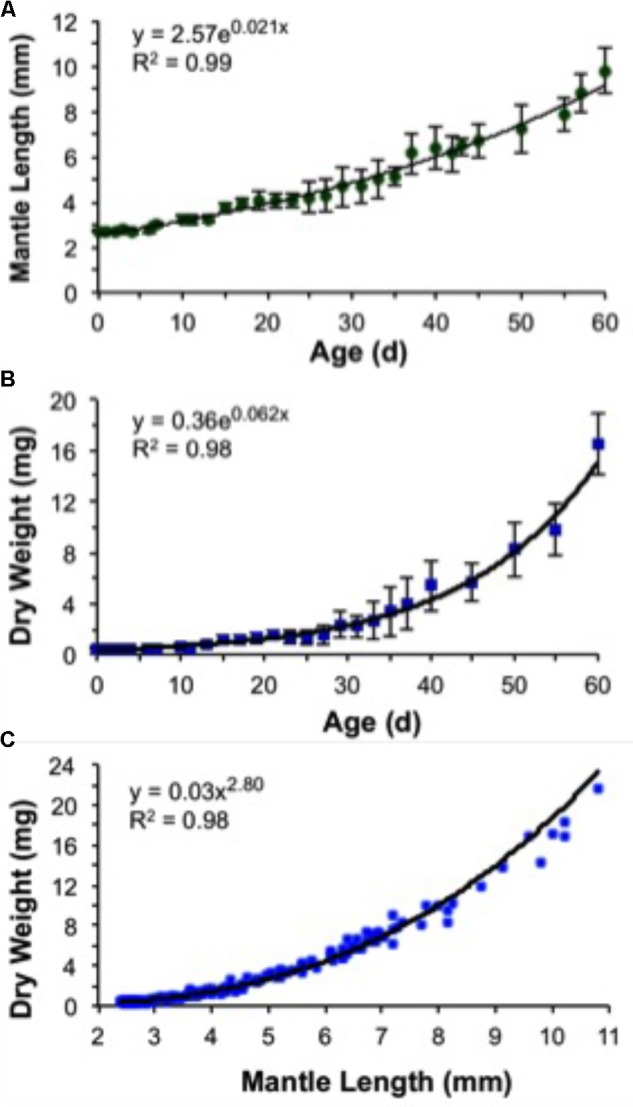
*Doryteuthis opalescens*. Growth rates of paralarvae reared for 60 days after hatching at 16 ± 1°C. **(A)** Mantle length versus age (values are means of 5–7 paralarvae ± SD), **(B)** dry weight versus age (values are means of 5–7 paralarvae ± SD), and **(C)** dry weight versus mantle length (*n* = 185).

A decrease in body mass took place soon after hatching due to the exponential rate of yolk utilization. As a result, 5-day-old paralarvae lost 13% of their hatching DW and showed the highest GR in MW, 1.81% day^-1^ (**Figure 10** and **Table 3**). The weight loss was regained over the next days. Thus, no significant increase in weight was observed until day 10 (**Figure 10B**). This represents the no net-growth phase that lasted approximately 10 days at 16°C (Vidal et al., 2002a). By day 15, paralarvae were almost threefold heavier than the newly hatched squid with the highest relative GRs for ML and DW for the study (**Table 3**). At day 40, MW had the lowest relative growth (**Table 3**), indicating that the proportion of MW to ML was decreasing, as the mantle was growing more slender. The second highest period of relative GRs occurred at day 60. At this time, the squid were growing more rapidly in length and weight (**Table 3**). After the no net-growth phase, growth was exponential at a rate of 6.2% body DW day^-1^ and 2.1% ML day^-1^ (**Figures 10A,B**). Mean body DW reached 16.5 mg at day 60 (**Table 3**). Thus, during the first 60 days of life, *D. opalescens* hatchlings double their mean DW five times and mean ML twice, at 12 and 31 days, respectively (**Figure 10**). The parameters from the length–weight relationships were *a* = 0.03 and *b* = 2.80, indicating slightly negative allometric growth (*b* < 3; **Figure 10C**).

## Discussion

### Swimming Ability of Paralarvae and Implications for Dispersal

This study documents the progressive development of swimming abilities in *D. opalescens* paralarvae as they undergo complex morphological, behavioral, and ecological changes that enable them to swim in schools. We detailed the transition from the pulsed jet-and-sink motion of paralarvae to the burst-and-coast swimming of juveniles. This significant improvement of swimming ability occurs within the first 2 months of life. Swimming faster and with more control co-occurs with growth (rapid increase of ML and fin size, changes in mantle muscle morphology; Preuss et al., 1997; Thompson and Kier, 2001, 2006), social interactions, and cognition capabilities. These three factors lead to the shift in paralarvae physiological ecology from plankton to nekton. Most importantly, the ecological implications of the swimming performance of paralarvae convey that they are competent to exert influence over fine-scale distribution and population dispersal by active swimming.

During ontogeny, an important factor changing as paralarvae increase in size is their hydrodynamic environment and the varying effects of viscosity and inertia expressed in the Re number. Hatchlings have bell shape (the highest MW/ML ratio), rudimentary fins when compared to juveniles and large yolk reserves (40–60% of their body dry weight; Vidal et al., 2002a) that restrict the volume of water held in their mantle cavities. Therefore, they are almost completely dependent upon constant short pulsed jet for locomotion (Bartol et al., 2009). The shape and size of hatchlings and their limited swimming ability are ideal traits for exploiting passive transport and dispersal by currents.

After yolk is fully absorbed, jetting is improved as paralarvae can hold proportionally more water in their mantle cavities; simultaneously predatory behavior (Vidal et al., 2002a), social interactions, and escape responses are intensified. The expression of these crucial activities results in more horizontal displacements and often demand burst SS. Average cruising speeds of paralarvae are within intermediate Re numbers (10 < Re < 200), in which both viscous and inertial flow forces play important roles. However, we have shown that >6 mm ML squid move in the inertia-dominated realm during escape jets (i.e., the zone of Re >200, reaching Re >3200).

A major change in shape and swimming performance of paralarvae takes place between 15 and 40 days post-hatching. Squid >40 days (>6 mm ML) develop the adult rocket-shaped body and are able to attain higher speeds. Indeed, average and maximum speeds increased considerably with age and size, allowing late paralarvae to cover short horizontal distances at higher speed (562 mm s^-1^). This substantial improvement of swimming performance allows paralarvae to occasionally escape from the intermediate Re realm during burst speed and operate under different flow regimes, suggesting that morphological changes in body shape incur in swimming performance. Late paralarvae high-speed escape ability should provide squid significant ecological advantage in terms of evading their predators.

We have demonstrated that swimming abilities of squid develop early in life, emphasizing that the passive drifting dispersal period of paralarvae is brief, as they form schools at 35–40 days of age (>6 mm ML). Studies have shown that *D. opalescens* paralarvae migrate to the surface in the first 6 h after hatching (Sidie and Holloway, 1999) and perform vertical diel migrations from 30 m (day) to the surface (night) by 14 days (Zeidberg and Hamner, 2002). The highest abundances of paralarvae are found in the neuston layer at night associated with cooler SST (13–16.5°C; Koslow and Allen, 2011; Van Noord and Dorval, 2017). Our experimental results are consistent with field studies and attest that paralarvae can actively influence their fine-scale distribution by migrating vertically, becoming aggregated in areas of high food availability and adjust their dispersal patterns in the field.

### Survival, Growth, and Swimming Performance

During the first 60 days of this study, paralarvae grew from 3 to 10 mm ML and doubled their DW five times (a near 40-fold increase). GRs were obtained at a rearing temperature of 16°C and would change depending on the mean temperature experienced by paralarvae in nature. Survival was the highest ever registered during any rearing experiments with loliginid squid (Yang et al., 1986; Vidal et al., 2002b). However, two main points of mortality were observed at 10 and 40 days after hatching; the first was related to the transition to exogenous feeding and the second possibly caused by the need for larger prey types (Vidal et al., 2002b).

The swimming performance and the motion paths of paralarvae changed considerably with development. Hatchlings drift with the current, exhibiting a more random motion and no coordinated swimming with nearby squid. During development, the interplay between fins, mantle contractions, and funnel action of paralarvae shifts from a physiology evolved for aerobic depth maintenance to inertia based gliding with the occasional need for anaerobic bursts of speed (Bartol, 2001; Thompson and Kier, 2001). There is a clear transition in swimming behavior with the ability to perform sustainable swimming and to school.

The large size of the fins relative to the ML in >40-day-old paralarvae promote enhanced swimming control necessary for the synchronized and fine-motor control swimming of schooling squid. Fins act as stabilizers and allow rapid braking by producing drag, generate lift at lower speeds to lessen negative buoyancy (Thompson et al., 2010; Bartol et al., 2016). The rate of fin beating nearly doubled between days 0 and 40, and then increased again by 27% by day 60, which occurs concurrently with significant changes in the motion pattern parameters and NND. Indeed, by 60 days, the swimming path has become finely controlled.

Fins are versatile – playing different roles depending on the swimming orientation and velocity. This was demonstrated in a study with adult *Lolliguncula brevis*, when squid are swimming backward in the tail-first position, fins function as stabilizers at low speeds, and propulsors at high speeds, while also providing net lift to hold the squid in a vertical position; but when squid are swimming forward arms-first, fins provide mainly lift and thrust to assist jetting (Anderson and Demont, 2005; Stewart et al., 2010). Squid are highly maneuverable and the coordination between jet and fins are the primary drivers of turning performance, which are involved both in prey capture and escape behavior (Jastrebsky et al., 2016). Accordingly, our results have shown a progressive development of fins during ontogeny, demonstrating that fin development is a requisite for sustained swimming and dynamic stability (Stewart et al., 2010) and the synchronized and maneuverable swimming of schooling early juveniles.

The SS obtained in the present study were similar to some other studies of cephalopod paralarvae, with average speed of 5–11 mm s^-1^ and maximum speed reaching 560 mm s^-1^. Maximum speed in *Octopus vulgaris* increased from 79.7 mm s^-1^ in hatchlings (2.0 mm ML) to 456.7 mm s^-1^ in 30-day-old paralarvae (4.5 mm ML) and then decreased when they were close to settlement (Villanueva et al., 1996). *Loligo forbesi* hatchlings (3.7 mm ML) can swim at average speeds of 6–20 mm s^-1^ and are able to attain a maximum speed of 250 mm s^-1^ (Zuev, 1964), while those of *L. vulgaris* can reach 160 mm s^-1^ in backward jets (Packard, 1969), and *D pealeii* (1.8 mm ML) have an average speed of 8.3 mm s^-1^ (Zakroff et al., 2018), but can reach up to 30.5 mm s^-1^ (Bartol et al., 2009). Ommastrephid hatchlings are among the smallest of the cephalopods; however, *Illex illecebrosus* (∼1.2 mm ML) and *Dosidicus gigas* can attain average and maximum speeds of 10 and 50 mm s^-1^, respectively (O’Dor et al., 1986; Staaf et al., 2008). By contrast, large hatchlings such as *Sepioteuthis lessoniana* (6.0 mm ML) are able to attain higher average speeds (60–150 mm s^-1^) during the first 2 months of life (Sugimoto and Ikeda, 2012).

### Swimming Performance of Starved Paralarvae

Starved paralarvae progressively lost the ability to swim (**Table 2**). Paralarvae starved for 48–72 h did not perform escape jets and behavior regressed to that similar to newly hatched paralarvae with short swimming paths and an inability to overcome a current (**Table 2**). Paralarvae were able to recover from 48 and 72 h of starvation with a survival rate of 60 and 37%, respectively after 8 days (Vidal et al., 2006). These results emphasize the importance of nutritional condition, as well as yolk reserves at hatching on studies of swimming behavior. Most importantly, they suggest that depending on the duration of the starvation period, unnourished squid would not be able to keep pace with schooling squid and thus would be at higher threat of predation without the protection of the school.

### Formation of Schools and Ecological Implications

Schooling was first noticed in 35- to 45-day-old paralarvae (6–7 mm ML). It is noteworthy that schools were always formed against the current, near the site of the inflow of water, where the currents were at their maximum velocity. This provides evidence that paralarvae might be capable of sensing flow (current shear) and are attracted to it, or their natural swimming pattern caused them to become aggregated in the highest gradients of flow. Loliginid squid have analogs of lateral lines on their heads and arms that contain epidermal hair cells that directly respond to sinusoidal water movements, which correspond to the sensitivity of fish lateral lines (Budelmann and Bleckmann, 1988) and ablation of these receptors increase the chance of predation in dark environments (York and Bartol, 2014).

Squid paralarvae are negatively buoyant (Sidie and Holloway, 1999; Martins et al., 2014), as a result, paralarvae sink when swimming stops and must constantly jet to stay suspended and this is energetically costly. Forming schools early in life in areas of gradients of flow (vertical turbulent mixing) could assist paralarvae in holding position, minimizing the energy allocated to jetting and aggregate them with their food in upwelling areas (Zuev, 1964; Koslow and Allen, 2011; Van Noord and Dorval, 2017). Indeed, *D. opalescens* paralarvae have been found aggregated within cyclonic gyres and eddy-induced upwelling in the Southern California Bight, where vertical mixing occurs at the frontal zone of warm and cold waters (Zeidberg and Hamner, 2002).

Schools were formed positioned against the current, which seems advantageous, as planktonic prey drifting with the current are brought toward the arms of squid positioned side-by-side. This suggests that there may be a close interplay between swimming performance, flow gradients, and foraging tactics of juvenile squid, representing a highly interesting topic for future investigations.

Yang et al. (1986) reared market squid through their lifecycle in larger tanks with higher current speeds. These researchers first noticed the ability to hold position at 40–45 days (10 mm ML) and to form schools by days 60–80 (15 mm ML). Our tanks had lower flow rates (1.0 cm s^-1^), thus the earliest possible school formation seems to be determined by size and the local currents, both related to sustained swimming ability (position holding). Nevertheless, there is a similarity between the sizes at which both *D. opalescens* and *Illex argentinus* form schools to forage. An exceptionally high density of *I. argentinus* juveniles of ∼10 mm ML was found associated with elevated plankton production in an upwelling area off southern Brazil (Vidal et al., 2010). This highlights the importance of size, rather than age, in the ability of squid to swim against a current and to form schools. Interestingly, by evaluating the effects of mantle and funnel aperture in a theoretical model of squid jet propulsion, Staaf et al. (2014) have proposed that the maximum efficiency of squid jet propulsion is found at 10 mm ML, which is a ML slightly larger than the ML size at which both *I. argentinus* and *D. opalescens* start to swim in schools. As the capability to rear squid improves the next step would be to study the key transition that occurs at 20–30 mm ML (Vidal, 1994; Moltchaniwskyj, 1995; Zeidberg, 2004). This is probably the point where squid attain a size that affords them inertial dominated movement for cruising speeds and a shift to a fish diet (Karpov and Cailliet, 1979).

### Swimming Behavior and Social Interactions

Changes in paralarvae swimming pattern and ability during ontogeny were accompanied by changes in social interactions associated with the formation of schools. These interactions provided insights into how squid acquire complex cognitive and fine-motor skills during ontogeny. Early paralarvae (0- to 15-day-old) swim with a relatively large NND and display aggressive behavior toward other paralarvae swimming nearby. Aggressive behavior and NND decrease progressively with growth. When paralarvae grow to a size that allows sustained swimming, they hover in place longer and often assume a similar body orientation as close neighbors. The close proximity and the parallel orientation soon promote enhanced polarization and synchronization, culminating with the formation of schools. These findings are consistent with the observations of Sugimoto and Ikeda (2012) for reared *S. lessoniana.* In the present study, however, the first squid to form schools swim in a more vertical orientation, perhaps a reflection of the slow current speed and tank configuration. Nevertheless, when the schools dispersed due to any disturbance, juveniles swam backward horizontally (lower angles) at higher speeds.

Another important feature of *D. opalescens* schools is that although more than one school was observed in a tank, each school was size assorted and the largest squid were frequently at the front, similar to the hierarchical structure observed in adult *S. lessoniana* (Boal and González, 1998). This hierarchical social organization underscores enhanced learning and cognitive capabilities (Ikeda, 2009; Sugimoto and Ikeda, 2012; Oshima et al., 2016). Indeed, social feeding interactions are often observed before the formation of schools in same-aged but different sized paralarvae. Large paralarvae frequently capture larger prey, which cannot be enclosed within the arms, facilitating kleptoparasitism (**Figure 6**). This social interaction provides smaller paralarvae the possibility of feeding on large prey items that they could not capture alone and that large paralarvae would not ingest entirely, maximizing resources (Vidal and Boletzky, 2014). Furthermore, this behavior denotes enhanced foraging repertoire based on learning through social interactions and could, ultimately, determine the hierarchical social organization of schools; large faster growing squid are the first to perform sustainable swimming and others follow. Kleptoparasitism is also common during rearing of *O. vulgaris* paralarvae (Vidal, EAG pers. obs.) and was recently reported for captive *Todarodes pacificus* schooling adults (Vijai et al., 2017). Nevertheless, field studies are required to provide a better understanding of this behavior.

Hunting behaviors develop in paralarvae in concert with changes in growth, muscle structure, swimming refinement, and social interactions. Paralarvae learn to catch copepods by honing predatory behaviors with a large increase in mortality at each developmental transition (Chen et al., 1996). It is perhaps significant that the adult-like tentacular strike behavior is only employed in prey capture in squid older than 40 days (Chen et al., 1996; Kier, 1996). The expression of this important behavior correlates well with our results on fin GR relative to the ML (**Table 3**), the ability to form schools and attain SSs that corresponds to Re >3200.

The progressive development of swimming abilities and social interactions described here may enable squid to accelerate learning, orientation, and cognition by amplifying fundamental social information from an early life stage. Morphological, neurophysiological, and sensory capabilities are required to perform parallel synchronized swimming with nearest neighbors while schooling (Pitcher and Parrish, 1993; Oshima et al., 2016). Earlier studies with fish have shown that vision along with the lateral line are of major importance for maintaining a particular position and orientation with respect to neighbors, while the lateral line has a key role in monitoring SSs and direction of other fish in the school (Partridge and Pitcher, 1980; Pitcher and Parrish, 1993).

The highly coordinated motion of juvenile squid observed in the present study during school formation, when the school scatters due to a disturbance and then reassembles, represents a rapid assimilation of group information. Schooling squid are constantly synchronizing and fine-tuning their behavior to what their neighbors are doing. Development of group dynamics from individual social interactions is crucial to the understanding of the mechanisms of social behavior. A recent study has shown that schooling decisions and group behavior in *D. pealeii* are influenced by the presence or absence of injured individuals (Oshima et al., 2016). Social interactions early in life may serve important adaptive functions of squid schools (Adamo and Weichelt, 1999; Sugimoto and Ikeda, 2012; Sugimoto et al., 2013).

## Conclusion

This study documented key events of survival and growth during early ontogeny of *D. opalescens*. Paralarvae undergo major and complex morphological, behavioral, and ecological changes during their first month of life, which may be requisites for schooling behavior. Our results revealed a progressive development of swimming abilities in squid from the random jet-and-sink swimming pattern of hatchlings with large NND to the finely controlled, parallel, and synchronized movements of schooling early juveniles at shorter NND. We have also shown that the feeding condition influences the swimming performance and behavior of paralarvae. In addition, the high relative GRs of mantle and fins in >15-day-old paralarvae lead to transformation in body shape (from a bell to a rocket) and enhanced SS and control, suggesting that morphological changes incur in swimming performance, enabling squid to perform sustained swimming. This event represents a key ecological and behavioral transition that occurs at about 6 mm ML and correlates well with the ability of paralarvae to reach high Re (>3200) during escape jets, transitioning to the inertia-dominated realm and from plankton to nekton. The main features of *D. opalescens* schools formed at an early age (35- to 45-day-old) were their hierarchical configuration (with the largest squid swimming at front), site of formation (at the highest gradients of flow inside the tanks), and positioned against the current. This provides evidence that paralarvae might be capable of sensing flow and deserves future investigations to improve our understanding of their sensory capabilities and fine-scale distribution. The results also suggest that the passive drifting period of squid is brief as paralarvae are competent to control their distribution and dispersal just after their first month of life. Social interactions prior to and during schooling provided insights into how squid acquire sophisticated cognitive and fine motor skills during ontogeny. Formation of schools at an early-life stage seems to be an adaptation for optimizing energy employed in foraging tactics and amplifying social information, thus serving important ecophysiological functions.

## Author Contributions

EAGV conceived and designed the study, conducted the experiments, collected and analyzed the data, and drafted the article. LZ helped with data analysis to draft the article and made improvements to the article. EB conceived and designed the study and helped with data analysis. All authors worked together to interpret the findings and approved the final version.

## Conflict of Interest Statement

The authors declare that the research was conducted in the absence of any commercial or financial relationships that could be construed as a potential conflict of interest.

## Abbreviations

FW: fin width
GR: growth rate
ML: mantle length
MW: mantle width
NGDR: net to gross displacement ratio
NND: nearest neighbor distance
RCD: rate of change of direction
Re: Reynolds number
SS: displacement swimming speed
